# Staffing at Ambulatory Endoscopy Centers in the United States: Practice, Trends, and Rationale

**DOI:** 10.1155/2018/9463670

**Published:** 2018-09-13

**Authors:** Deepak Agrawal, Rajeev Jain

**Affiliations:** ^1^Department of Medicine, Division of Digestive and Liver Diseases, University Texas Southwestern Medical Center, Dallas, TX, USA; ^2^Texas Digestive Disease Consultants, Dallas, TX, USA

## Abstract

**Background:**

Endoscopy nurse (RN) has a pivotal role in administration and monitoring of moderate sedation during endoscopic procedures. When sedation for the procedure is administered and monitored by an anesthesia specialist, the role of an RN is less clear. The guidelines on this issue by nursing and gastroenterology societies are contradictory.

**Methods:**

Survey study of endoscopy lab managers and directors at outpatient endoscopy units in Texas. The questions related to staffing patterns for outpatient endoscopies and responsibilities of different personnel assisting with endoscopies.

**Results:**

Responses were received from 65 endoscopy units (response rate 38%). 63/65 (97%) performed at least a few cases with an anesthesia specialist. Of these, 49/63 (78%) involved only an endoscopy technician, without an additional RN in the room. At 12/49 (25%) units, the RN performed tasks of an endoscopy technician. At 14/63 (22%), an additional RN was present during endoscopic procedures and performed tasks not directly related to patient care.

**Conclusions:**

Many ambulatory endoscopy units do not have an RN present at all times when sedation is administered by an anesthesia specialist. An RN, when present, did not perform tasks commensurate with the education and training. This has implications about optimal utilization of nurses and cost of performing endoscopies.

## 1. Introduction

Endoscopies at the ambulatory surgical centers (ASCs) are well-established models of care offering convenience, efficiency, and economy for low-risk endoscopies. For ASCs to be successful, it is important to keep quality and patient satisfaction high and costs low. Optimal staffing of the endoscopy units is, therefore, essential. The endoscopy team usually consists of a registered nurse (RN) who administers moderate sedation and an endoscopy technician who assists with technical procedural-related activities such as taking biopsies and removing polyps. The person performing the tasks of a technician may be another RN or a trained unlicensed person. In the last few years, more and more endoscopists prefer sedation with propofol, which is faster and shorter acting [1]. Propofol sedation qualifies as deep sedation since spontaneous breathing can be affected and per regulations in the United States can be administered only by an anesthesia specialist. This raises questions about the role of an RN during endoscopic procedures since their main responsibility of administering sedation is now taken over by an anesthesia specialist. Endoscopy units outside United States have different staffing models but have the same concerns about costs and efficiency of health care.

The American Society of Gastrointestinal Endoscopy (ASGE) guidelines state that when an anesthesia specialist is responsible for sedation [2], an additional RN is not needed while the Society of Gastroenterology Nurses and Associates (SGNA) recommends the presence of an RN to assist the endoscopy team [3]. These contradictory statements are not supported by any references or data. The practice patterns, thus, have emerged based on opinions, perceptions, needs, and wants. With increasing health care costs and decreasing reimbursements for procedures, optimal resource utilization has become an important issue [4]. Furthermore, there is a significant shortage of RNs in the United States and optimal utilization of their knowledge and expertise is important.

We conducted a survey of ambulatory surgical centers (ASCs) and endoscopy units in Texas, United States, to determine the practice patterns regarding type of sedation (moderate vs. deep), staffing of endoscopy units, and the role of RNs during procedure when sedation is administered by an anesthesia specialist.

## 2. Methods

A questionnaire was constructed after open-ended interviewing of gastroenterologists and nurses from two institutions. The responses were reviewed by additional gastroenterologists and nurse managers from three different institutions, not included in the study, for linguistic, internal, and external validation of the questionnaire. An online version of the survey was created using https://www.surveymonkey.com/. The questions asked included type of endoscopy unit (ambulatory versus hospital based); number of endoscopic procedures performed per month; type of procedures; percentage of patients with different American Society of Anesthesiologists (ASA) classification; method of sedation; and personnel present in the endoscopy room during procedure and their responsibilities during procedure.

The names of ambulatory surgical centers performing endoscopies were obtained from the Texas Department of State Health Services [5]. An email with hyperlink to the survey was sent to the managers or directors of the endoscopy units. If no response was received after one week, the endoscopy units were contacted with a phone call to make sure they had received the survey. If email addresses were unavailable, the survey was faxed to the endoscopy unit. All data were obtained anonymously. This research was granted exemption by the Institutional Review Board of the University of Texas Southwestern Medical Center.

Data were summarized using descriptive statistics. Response rates were calculated as number of surveys with responses divided by number of surveys distributed. All the data supporting the results are shown in the paper and can be applicable from the corresponding author.

## 3. Results

The surveys were distributed to 172 endoscopy units and responses received from 65 (response rate 38%). Details of the ASCs are shown in Table 1.

### 3.1. Use of an Anesthesia Specialist

Overall, 63/65 (97%) of endoscopy units performed cases with an anesthesia specialist. Of these, 30 (46%) endoscopy units performed 100% of their procedures and 15 (23%) performed 75–99% of their procedures with deep sedation administered by an anesthesia specialist. Two endoscopy units performed their cases only with moderate sedation and did not use an anesthesia specialist.

### 3.2. Use of RNs

Of the endoscopy units that performed their cases with an anesthesia specialist, 49/63 (78%) involved only an endoscopy technician, without an additional RN in the room. At 12/49 (25%) endoscopy units, the RN performed tasks of an endoscopy technician. The other endoscopy units had a “floating nurse” who was available, if needed.

At 14/63 (22%), an additional RN was present during endoscopic procedures and performed tasks not directly related to patient care. The responsibilities of RNs included at least one of the following: performing a time out before the procedure, documenting endoscopic accessories used, and documenting/labeling pathology samples. At some units, RNs had additional responsibilities. For example, at 7/26 (27%) endoscopy units, the RNs helped with consents before the procedures. At 2/26 (8%) endoscopy units, the RNs completed the preliminary endoscopy report or discharge instructions after discussions with the endoscopist.

Twenty-six endoscopy units had at least one RN present during endoscopy, when sedation was administered by an anesthesia specialist (12 endoscopy units had 1 RN and 14 units had 2 RNs). The reasons given by endoscopy units for having an RN along with anesthesia specialists included regulatory requirement to have an RN in the room (18/26, 69%), complete documentation requirements (17/26, 65%), improved patient safety (4/26, 15%), and need for an RN for submucosal injections (3/26, 12%). At the 37 endoscopy units without an RN, submucosal injections were mainly performed by the technician assisting with the procedures except at 7 (19%) units where a floating RN was called, as needed. At two endoscopy units, the technician was allowed to assist with submucosal injection of saline and dye but not diluted epinephrine.

## 4. Discussion

ASCs avoid urgent circumstances and high-risk patients and are especially well suited for care-process standardization. Reducing variation and standardizing workflows that allow staff to work at their level of training and scope of practice is necessary to provide high quality, cost-effective care. Conflicting statements from professional societies or ambiguous statements from credentialing and regulatory bodies can create confusion about standards of practice. We highlight such an issue in our study, which although focused on endoscopy is applicable to other procedures at ASCs.

What should be the appropriate staffing during endoscopy when endoscopy sedation is provided by an anesthesia specialist? What should be the role of an RN in this situation? The American Society of Gastrointestinal Endoscopy states, “When endoscopy is with an anesthesia specialist, one endoscopy staff member is required to assist the endoscopist with the technical portion of the procedure. This person may be an unlicensed active personnel (UAP), licensed practical nurse (LPN) or an RN. The presence of an RN is not mandatory in this setting” [2, 6]. The Society of Gastroenterology Nurses and Associates in their minimum RN staffing position statement states, “When an anesthesia specialist is providing the sedation, the RN will remain in the procedure room to assist the healthcare team” [3]. The contradictory position statements are confusing for physicians, nurses, management of endoscopy units, and accreditation organizations and introduce variation in standards of care and resource utilization.

The issue highlighted in our study, while focused on endoscopy, has a more generalized appeal for other procedures at ASCs. Conflicting statements from professional societies or ambiguous statements from credentialing and regulatory bodies are not uncommon. Our survey is the first study, to our knowledge, to determine how ASCs choose to staff their endoscopy units and the rationale for their approach. Our results show that, when an anesthesia specialist administers sedation, less than one fourth of the ambulatory endoscopy units have an RN present during the procedure. Most of them instead have a “floating” nurse who is available to help as needed. Notably, the change in staffing model of not having an RN in the room appears to be a recent trend. ASGE survey from 2013 had reported that in ambulatory endoscopy units, an RN was present in the room along with anesthesiology provider 62% of the time [7]. We speculate that this may be in response to decreasing reimbursements and emphasis on optimizing resource utilization [8].

The responsibilities of an RN, when present in the room along with an anesthesia specialist, mainly included performing a surgical time out, documenting endoscopic accessories used (for billing purposes), and/or helping with pathology samples—none of which require a person to have nursing knowledge or expertise. When an RN was not present, these tasks were completed by the endoscopy technician and/or anesthesia specialist. The increasing documentation requirements and paperwork sometimes necessitates an additional person in the room, other than a technician. Some endoscopy units used the floating RN or another technician for these tasks.

One of the arguments of having an RN in the room along with the anesthesia specialist is increased patient safety. The premise of such an argument would be that deep sedation by an anesthesiologist has more complications compared to moderate sedation by an RN and that an extra RN is needed helpful to respond to these complications. There is no data to support these premises. Before the current regulation that administration of propofol can only be performed by an anesthesia specialist, RNs administered propofol under the endoscopist's supervision. This was referred to as nurse-administered propofol sedation (NAPS), the safety of which has been well established in many prospective and retrospective studies [9, 10]. Notably, in these studies, no additional RN was present.

Another reason why endoscopy units need RNs during the procedure is the belief that regulations require that submucosal injection of saline, epinephrine, and dyes into the gastrointestinal wall can only be performed by a nurse and not an unlicensed personnel (UAP) or a technician. The regulations on what licensed and unlicensed personnel are permitted to do during a procedure are determined by the state but the directives are often not stated clearly. The Joint Commission, the Accreditation Association for Ambulatory Health Care, and Centers for Medicare and Medicaid Services do not define the specific qualifications or number of staff required [11–13]. Rather, they generalize that the staff be adequate in number with appropriate training and supervision. In the state of Texas, a surgical technician can inject submucosal normal saline or dye as long as the technician is deemed competent to do so and performs tasks under continuous and direct physician supervision [14]. In our survey, many respondents believed that having an RN in the room along with an anesthesia specialist was per state or federal regulations and accreditation guidelines. Few respondents commented that they thought the use of an RN in addition to the anesthesia specialist was standard of care. Our survey shows that the majority of the endoscopy units in Texas do not have an additional RN in the room along with anesthesia provider and thus would be considered a standard of care [15].

The question if an RN should be present at all times during the procedure is important and especially relevant in present health care scenario where there is severe shortage of RNs and endoscopy units are under pressure to provide more cost-efficient care to the patients. A recent report by American Association of Colleges of Nursing estimates the nursing shortage to be 260,000 by year 2025 [16]. In our survey, few respondents specifically commented on having difficulty hiring RNs. Declining reimbursements, bundled payments, and reference pricing have now made it imperative to reevaluate the costs of endoscopic procedures [4, 8, 17, 18]. An ASGE survey of endoscopy units in the US reported that clinical full-time equivalents (FTEs) in one endoscopic procedure have increased from 4.1 in 2008 to 4.7 in 2011—a 15% increase while the reimbursements have decreased. Clinical labor represents more than 40% of the total endoscopy unit cost [7]. More importantly, patients now bear the burden of increased costs due to high deductible plans or copays.

When an anesthesia specialist administers sedation, RNs present in the room did not perform tasks commensurate with their training and experience. Endoscopy nurses are a valuable resource and their knowledge and expertise can be utilized in more important ways. For example, the endoscopy nurses can be helpful in tracking quality indicators of endoscopy. The endoscopy nurse can also help improve communication about endoscopic procedures with the patients. In a recent study, patients at a safety-net hospital, who gave informed consent for an endoscopy after an educational class given by nurses, reported being better informed about the procedure compared to patients who were consented in the gastroenterology clinic [19].

Our study has limitations. First, the study was conducted in the United States and may not be applicable to endoscopy units in other countries, which have different regulatory requirements. Second, our study population was restricted to ambulatory endoscopy units, which affects generalizability of the results to endoscopy units within a hospital. This was intentional to ensure homogeneity of patient population across different endoscopy units. For example, the patients were mostly ASA I and II and less likely to include complex indications. Second, we surveyed only endoscopy units in the state of Texas. We did so to avoid regional variations in policies and regulations regarding scope of practice for surgical technicians, LPNs, and RNs. Fourth limitation is the possibility of information bias. Response bias could have occurred if endoscopy units that do not use an RN in the endoscopy unit were more likely to respond. However, we believe that our data is more representative of the current trends than nonresponse bias. A 2012 survey of endoscopy practices conducted by ASGE showed that nationwide 66% endoscopy units use propofol for sedation which is very similar to our data. Even, if there is some response bias, our survey shows the feasibility and safety of procedure with an anesthesia specialist without an RN in the room. Finally, our survey does not address the issue of safety of endoscopic procedure with different staffing models.

In conclusion, our study highlights different endoscopy staffing models and how different endoscopy units are adapting to the changes in health care—increasing demand for the more expensive deep sedation, decreasing reimbursements and nursing shortage. Interests and safety of the patients come first and endoscopy units should ensure that the staff assisting with the procedures are well trained. Most ambulatory endoscopy units now do not have an RN present at all times when sedation is administered by an anesthesia specialist. This staffing model meets the regulatory requirements in the US, is not associated with any adverse outcome, and may allow more appropriate utilization of endoscopy nurses.

## Figures and Tables

**Table 1:** Characteristics of endoscopy units surveyed.

	Range	Mean
Number of procedure rooms	2–5	3.4
Number of procedures performed in a month	220–1350	796
Percent of procedures that are		
Upper endoscopies	20–50%	32%
Colonoscopies	50–90%	68%
Others	0–3%	<1%
ASA classification of patients		
ASA 1	20–35%	24%
ASA 2	25–60%	41%
ASA 3	15–45%	35%
ASA 4	0-1%	<1%
Percent of procedures in an endoscopy unit with an anesthesia specialist	0%–100%	72%

## Data Availability

All the data supporting the results are shown in the paper and can be applicable from the corresponding author.

## Additional Points

*Biostatistics Statement*. Simple descriptive statistics was used.
